# Medical Education 4.0: A Neurology Perspective

**DOI:** 10.7759/cureus.31668

**Published:** 2022-11-19

**Authors:** Zaitoon Zafar, Muhammad Umair, Filzah Faheem, Danish Bhatti, Junaid S Kalia

**Affiliations:** 1 Neurology, NeuroCare.AI Neuroscience Academy, Dallas, USA; 2 Movement Disorders, University of Central Florida College of Medicine, Orlando, USA; 3 Neurology, NeuroCare.Al, Dallas, USA; 4 Neurology, NeuroCare.Al Neuroscience Academy, Dallas, USA

**Keywords:** virtual care, telemedicine, medical education, teleneurology, neurology

## Abstract

Medical education faces a difficult challenge today; an exponential increase in knowledge and the rise and rise of disruptive technologies are making traditional education obsolete. As the world nears the era of Industry and Healthcare 4.0, the medical community needs to keep up and prepare physicians for a hyper-connected digital world. Virtual neurological care is poised to be at the forefront of care delivery claims, yet the virtual communication of neurological knowledge is still in its infancy. This increasing digitalization of care and education is both an opportunity and a challenge. With this paper, the authors aim to bridge the gap between technology and neurological education. After a thorough review of recent literature and assessing current trends, the authors propose that contemporary medical education must adhere to the following tenets: Hybrid, Mobile, Mixed-reality, Open Access, Collaborative, Peer-reviewed, Intelligent, Game-based, and Global. We identify and align education objectives with the needs of future digital neurologists. The authors also discuss real-world advances that are aligned to serve the next generation of patients and providers.

## Introduction and background

We live in accelerated times. The era of radical digital transformation has brought about Industry and Healthcare 4.0 [1]. Additionally, the SARS-CoV-2 pandemic has resulted in irreversible changes to modern human life, including the delivery of healthcare and the pursuit of education. Even before the pandemic, education (including medical education) was undergoing a massive shift toward the online world, with Massive Online Open Courses (MOOCs) and the rise of new course platforms like Udemy, Skillshare, Udacity, etc., to name a few [2-4]. Established universities were forced to offer more of their curriculum online following lockdowns and safety measures, with many predicting that the delivery of higher education has now changed forever [5]. Unfortunately, medical education has been lagging in adapting to an increasingly digital world. This is unfortunate, considering the necessity of medical knowledge and the importance of its verification.

The medical community needs to prepare for a generation of mobile-first students, ceaselessly connected to the internet and constantly bombarded with information. An increasingly virtual world necessitates an overhaul of how medical knowledge is imparted, accessed, and learned. The field of teleneurology has prior experience in telemedicine delivery with its management of acute stroke care. Despite this advantage, the virtual communication of neurological knowledge and education is still in its inception.

This paper intends to review the current status of digital education concerning medical knowledge. Furthermore, the paper aims to identify gaps in the current system, in reference to Industry, Healthcare, and Education 4.0 The authors intend to provide a vision, a strategy, and some key guidelines for digital medical education with a focus on neurology. The advent of up-and-coming impactful ventures in alignment with the principles of Education 4.0 will also be discussed.

## Review

A virtual new world: Industry 4.0, Healthcare 4.0, and Education 4.0

As of January 2021, almost five billion people are connected via the internet [6]. We are now a digital population living in a shared virtual space. This is both a great opportunity and a daunting challenge. The pandemic has hastened the shift toward digitalization and virtualization. This forced change has led to an ever-lasting cultural shift where people prefer to work remotely and have healthcare and education delivered virtually [7,8].

Industry (4.0)

Industry 4.0 denotes a world where control and decision-making are decentralized, wherein various systems are integrated to achieve hyper-connectivity. The core concept of the 4th Industrial Revolution is the strengthening of the connection between the cyber and our lived, physical world. Healthcare 4.0 will also be a similarly hyper-connected, decentralized system. Table 1 defines the principles of Industry 4.0 and its application in Healthcare 4.0 and Medical Education 4.0 [1].

**Table 1:** Principles of Industry 4.0 and its application to Healthcare 4.0 and Medical Education 4.0

Principles of Industry 4.0	Healthcare 4.0	mEducation 4.0
Artificial Intelligence (AI)	Artificial intelligence is and will be used heavily in all domains of healthcare: imaging, diagnostics, genomics, and analytics.	Knowledge about the opportunities and limitations of artificial intelligence in clinical practice. Create learning systems with the help of AI to augment learning.
Mixed Reality (MR)	Virtual reality and Augmented reality infused with AI to improve patient outcomes	Learn to use this new medium to help a new generation of patients. Study human cadavers, practice surgical skills and procedures, learn about medical devices, and gain familiarity with clinical settings.
Intelligent Robotics	Intelligent robotics will be transformative for patients regarding medication adherence and rehabilitation.	Learn the opportunities and limitations of intelligent robotics in patient lives to improve care and health delivery.
Cybersecurity	Privacy and access are important in the digital world. Blockchain allows for secure but accessible access to data.	Understanding the digital underpinning of patient data privacy and cybersecurity
Internet of things (IoT)	Health IoT will transform how we monitor patients. These new sensors are getting more accurate and cheap.	The role of Health IoT for patients will continue to expand exponentially. Its use and limitations need to be evaluated by future physicians.
Integration/Collaboration/Cloud	All systems will converge and collaborate with an application programming interface (API).	Learning to take care in a digital collaborative environment. Understanding system limitations.
Digitization	We are increasingly moving towards creating digital twins of our patients where there is a virtual existence	Comprehend the concepts behind digital twins in healthcare and how they can improve care and create more opportunities for research
Virtualization	Virtual Care is already mainstream but will the whole continuum, including clinical trials, increasingly be virtualized	Learning how to provide care in a virtual setting and environment (Metaverse). Also, understanding decentralized virtual clinical trials

Healthcare (4.0)

Digital health is defined as the use of digital technologies to impart healthcare. Having been proven to improve the safety and efficiency of healthcare delivery, it is now becoming the new norm [9]. Virtual healthcare - where the patient and physician interact digitally in real-time - is poised to become a quarter-trillion-dollar industry, with neurology and psychiatry at the forefront of care delivery claims [10]. Digital technologies have modernized all facets of patient care, from diagnostics to therapeutic options. In 2018, U.S Food and Drug Administration (FDA) approved the first autonomous artificial intelligence (AI)-based device for diagnostics, in addition to virtual reality-based therapies. These advancements represent a significant paradigm shift in the delivery of healthcare [11-13].

Education 4.0

**Figure 1:**
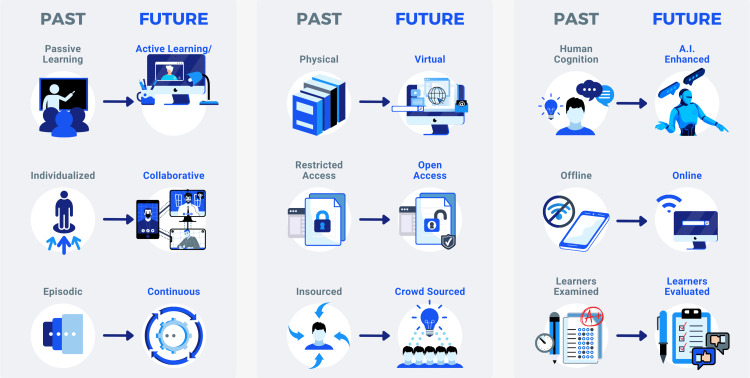
The past and future of education

Digital technologies have enabled education to be a continuous lifelong process (Figure 1). What was previously a one-way communication model between one-to-few (Education 1.0) has evolved to be one-to-many (Education 2.0). The current Education 3.0 is a symbiotic participatory continuous learning and evolving system. We are now nearing the era of Education 4.0, a learning system that aligns itself with the principles of Industry 4.0. 14 (Table 1). 

Sustainability and efficiency is the primary ethos of Industry 4.0, with personalized experiences - aided by AI - being of utmost importance. In Education 4.0, the evolution of collaborative, participatory learning with the help of new digital technologies like AI, mixed reality (MR), etc., will continue, enabling a more immersive learning experience [14].

Medical Education 4.0: hybrid, mobile, mixed-reality, open access, collaborative, peer-reviewed, intelligent, game-based, global

Medical knowledge doubles every 73 days [15]. Dr. Densen accurately predicted in 2011 what medical education is facing today; an exponential increase in knowledge and the rise of disruptive technologies are making traditional education obsolete. By the time medical students graduate, the knowledge they acquired during their early years becomes outdated.

Medical students of today are "digital natives," who have access to a wealth of information at any given moment, a "knowledge cloud" [16,17]. Therefore, a shift to meta-learning and "knowledge management" is imperative, as students need to be taught to discern between practical, verified, and inauthentic information.

The responsibility for disseminating vetted medical knowledge falls in the hands of the medical community. One of the issues brought to the forefront by the coronavirus disease 2019 (COVID-19) pandemic is the rise and spread of medical misinformation. Unverified and politicized medical knowledge is capable of causing civil unrest and societal division [18]. It is time to move toward medical education built on the principles of our new virtual world, as the age of AI is heralded in healthcare [19].

**Figure 2:**
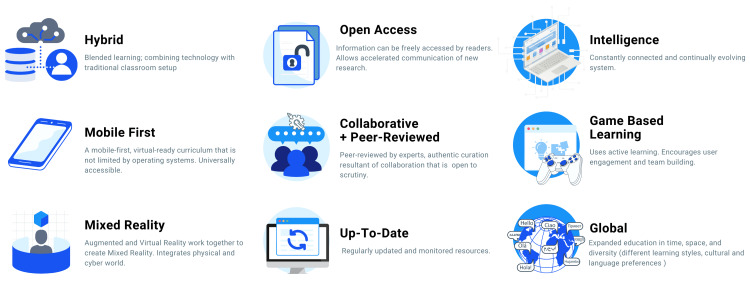
Tenets of Medical Education 4.0

For clinical neurology to keep up with the time and to produce competent digital neurologists, we have to prepare medical students for a cyber-physical, virtual new world. After a probing of recent literature and assessing current trends, the authors propose that contemporary medical education must adhere to the following tenets: hybrid, mobile, mixed-reality, open access, collaborative, peer-reviewed, intelligent, game-based, global (Figure 2).

Hybrid: Online and In-Person

Hybrid, or blended learning, is an approach to education that combines the technologies available for online learning with traditional classroom setups. This approach is proving itself to be the best of both worlds [20]. This brings the opportunity to create one's own learning experience, ease of access, and flexibility to medical students. Virtual clerkships have also been incorporated into preclinical medical education [21]. Virtual morning rounds aid in developing clinical and diagnostic thought processes and communication skills [22]. Furthermore, critical feedback from peers increases trainee engagement [23]. Neurology has been at the forefront of this, including but not limited to virtual neurological examinations and online neurocritical care education, to name a few [24,25]. Post-pandemic, this needs to be the norm and standard teaching practice globally. This standardization will move the needle from online learning to truly virtual learning.

Learning in a Virtual Environment

The logical evolution of hybrid education is its progression into a virtual environment. The key technological advancement lies in virtual reality (VR) and augmented reality (AR). VR is a computer-generated simulation of an environment that can be physically interacted with via special electronic equipment. AR enhances the physical world by bringing virtual objects into it and allowing users to interact with them. Both AR and VR work together to create mixed reality (MR), bridging the physical and virtual worlds [26].

VR is a revolutionary tool and has been deployed to understand anatomy [27]. According to a recent systematic review, VR is most commonly used in health-related education [28]. Neurosurgical simulation based on VR has made tremendous progress and has clear benefits [29]. Systems like VR-based neurological examination teaching tools (VRNET) are being developed and tested [30]. Virtual dissection can be performed on the real brain [31]. AR-enhanced tele-proctoring is also being used to train future neuroendovascular surgeons [32].

Mobile, Universal Design

Since 2017, mobile phones have transcended other devices to access the internet. Over 90% of all digitally connected people use mobile phones to access the internet, and it is anticipated that 72.6% of the world's population will access the internet via smartphone by 2025 [33,34]. Due to low financial and infrastructural constraints, many resource-limited countries have shifted to mobile-first internet populations. Through a curriculum design that is mobile-first and virtual-ready, the educational content developed is not limited by an operating system (OS) or device type, allowing it to be accessed universally.

Collaborative, Peer-Reviewed; Up-to-Date

As aforementioned, medical knowledge doubles every 73 days [15]. In order to account for this massive efflux of knowledge, resources with collaborative content editing users are our best bet. Wikipedia, a volunteer-based contribution system that is open source, has gone "head-to-head" with expertly developed resources like Britannica and Microsoft Encyclopedia [35]. This volunteer-based system is essential in the digital world as no single company or organization can keep up with the ever-changing and ever-growing knowledge base demand. Similarly, medical education needs to evolve with crowd-sourced, volunteer-based, and open-access systems. More than 20% of the world's population depends on online encyclopedias, such as WikiDoc, to get health-based information [36]. The main challenge is authenticity and trust. Systems must be developed to assure accuracy, as discussed below, by peer-review methods [37]. Authentic open-access resources will help curb the misinformation plaguing online medical content [38].

Open Access

The open educational resources (OER) movement began organically in the 2000s with the sole goal of making education accessible to all [39]. The medical community's answer to OER is Free Open Access Medical education (FOAM). OA provides innumerable benefits to the community and the authors. It is estimated that open-access articles receive 18% more citations [40], resulting in a greater impact for authors. OA is beneficial for societal innovation and progress [41], as it allows accelerated communication of new research [42]. The public benefits further from accessibility and having research translated into many languages without restrictions.

cOAlition S is an initiative to make full and immediate open access to research publications a reality. The idea is to have research and publications available for free and open access, especially research that is funded by public money [43].

Intelligent Just-in-Time Learning

As discussed, the onslaught of updates makes it difficult to keep up with medical knowledge. As therapeutics become increasingly digital, the rate and pace of change will be even steeper, requiring clinicians to evolve to meet the demands of new knowledge rather than the other way around [44]. Intelligent education systems can bridge this gap between knowledge and physicians.

Medical education is best delivered intelligently and accessible just in time, preferably embedded within our electronic health records systems. Modern search tools are extremely effective in retrieving or embedding these trusted educational resources. Such a system is constantly connected and continually evolving, as only these characteristics can help account for the massive efflux of data in medicine while being monitored remotely.

Education of Artificial Intelligence Applications In Healthcare

Artificial intelligence is poised to change healthcare for the better and become physicians' primary tools to deliver care [45]. Yet, despite the increasing integration of Big Data and AI into clinical practice, current knowledge of AI among healthcare workers is abysmally low. The number one reason for this is a lack of AI-related knowledge among medical school faculty and hence a resultant lack of incorporation into the medical school curriculum [46]. It is the medical community's responsibility to ensure the proper implementation of AI in enhancing patient care, and this can only be achieved if AI is incorporated into the medical education curriculum [47]. Medical students should, at the least, understand the fundamental concepts of AI and have sufficient knowledge of data science, biostatistics, and evidence-based medicine [48] to optimally use AI and create future applications for patient (and physician) care.

Artificial Intelligence-Assisted Medical Education (AIMED) - Automation, Intelligent Feedback, Virtual Teachers

Universities are increasingly employing AI to gauge their students' performance to guide them accordingly. The result is a very personalized self-tracking system with the sole purpose of informing the user to create an optimal learning routine. AIMED has the potential to help both students and teachers. It saves time for focused work, maximizes their efforts, and creates a more communal environment with teachers leading guided online discussions [49].

In a study where an AI-based medical image learning system was developed to assist students in identifying hip fractures, the group with AI-assisted learning scored considerably higher [50]. Another instance where AI-based problem-based learning was used in a clerkship in ophthalmology also reported an increased level of understanding from students [51]. This shows that AI can considerably shorten learning curves and augment medical education.

Game-Based Learning (GBL)

Another arena in the digitization of education is the application of game-playing elements (e.g., scoring points, rules of play, etc.) To encourage user engagement, i.e., gamification, game-based learning (GBL) uses active learning, and its effectiveness has been proven by multiple studies in various disciplines, ranging from mathematics to history [52]. Gamification of science education is not new; many programs have been established under the principles of GBL [53]. Similar programs have also been developed for the teaching of neurology. Gamified strategies to aid learning of the cranial nerves [54], a game of cards involving neurological symptoms played among two groups of a class in the second year of medical school [55].

GBL has also been used as a tool to assess and enhance the training of medical residents, with satisfactory results in augmented learning, knowledge acquisition, and team building, and has also resulted in the conservation of both time and money [56,57]. The uses of GBL do not stop at the graduate level but have also been applied to Continuous Medical Education (CME) in stroke [58]. Learners at all levels of medical education report greater satisfaction with the GBL experience than with traditional methods.

To the effects and advantages of gamification of medical education, researchers in Malaysia have proposed a framework for GBL in the science, technology, engineering, and mathematics (STEM) fields. Their article outlines the specifications required in a game to reach desirable results, from the phase of conception to execution and outcome measurement [59].

Learning as a Global Phenomenon

As noted, medical education needs a system that meets the requirements of the future. The digital and virtual tools, as discussed above, need to be incorporated. Given the cultural shift to virtual, medical educators need to accommodate different learning styles and cultural and language preferences. Special attention must also be paid to the neurodiversity of learners [60]. As proposed by Dr. Joshua Drew, technology should be used to "expand the classroom in space, time, and diversity" [61]. AI in learning systems is one of the most effective ways to incorporate learning systems that are evolving and global. This can be achieved using AIMED, as these tools work with big analytics and can be hyper-tuned for local requirements.

Advances in medical education made by traditional institutions

Institutes are incorporating digital education by utilizing the steps of Kern's Curriculum Design [62]. Following the example of the neurology residency program at Mayo Clinic, more and more training programs can implement the telestroke curriculum in their residency programs [63]. Programs can use components of observation, didactics, simulation, and real-life experience to formulate a teleneurology curriculum. In addition to this, programs can follow the footsteps of the University of California San Francisco by involving their residents in telehealth training programs for a hands-on experience [64]. Programs can send their residents to remote facilities with telestroke management to get competent in telestroke.

Advances in medical education beyond traditional institutions

Medical education resources in alignment with the aforementioned tenets are already gaining momentum. Here, the authors elaborate upon a few real-world examples. 

WikiDoc

WikiDoc is "an open-source website that allows an international community of healthcare professionals to add and edit medical content in a process termed co-creation" [65]. WikiDoc was established in 2006 on the framework of collaborative work applications, i.e., "wiki" [66]. WikiDoc has several strengths; a wide array of co-authors curtail bias, provide a neutral point of view, and offer extensive detail about topics poorly documented beforehand. WikiDoc reduces the chances of any factual errors occurring by being open access. Emerging and evolving topics are addressed very quickly, and they keep on evolving, with newer information added as the topic matures. WikiDoc does not allow the pushing of any viewpoint. The comment section on WikiDoc serves as a great place for voicing opinions and exchanging views, hence creating an interactive space. WikiDoc is based on an important principle of 'Copyleft,' which is an idea that ensures that copyright information is readily available for examination and modification [67].

Neurologypocketbook.com

**Figure 3:**
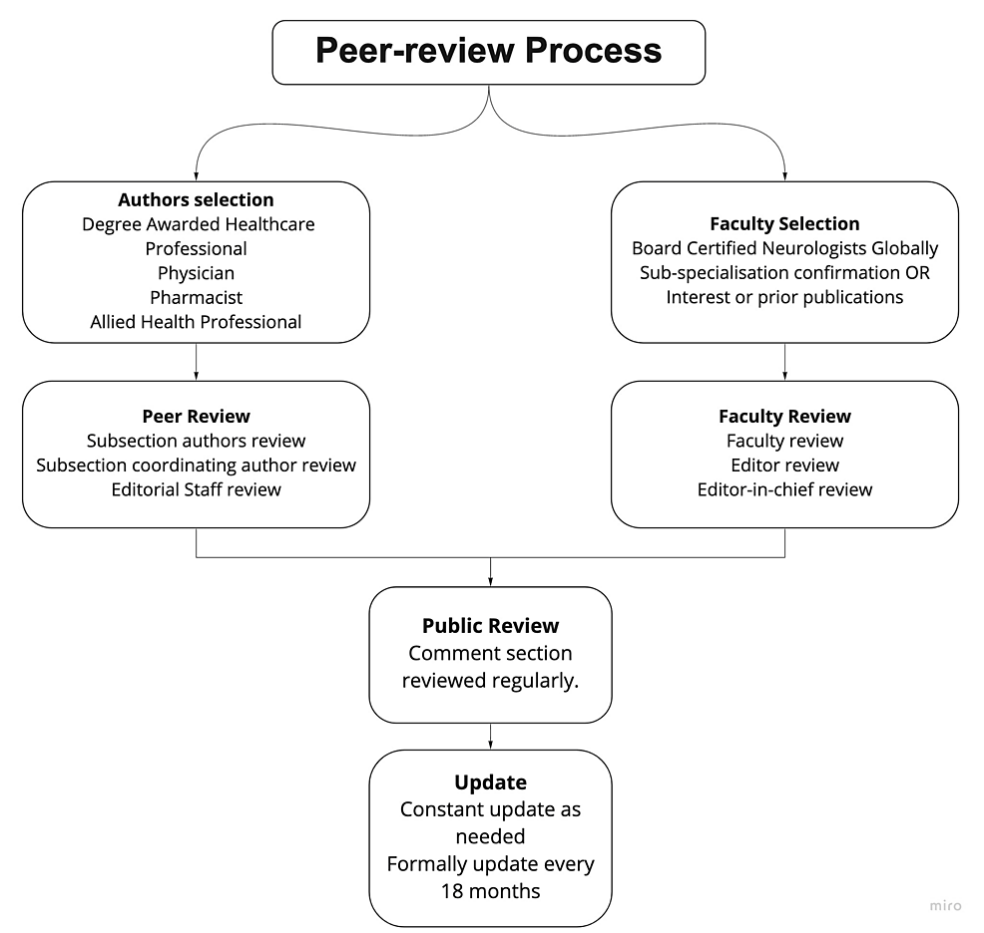
Peer review process of neurologypocketbook.com

eurologyPocketBook.com is an "Applied, Concise, Practical, Up-to-date, Mobile-friendly & free access Pocketbook of Neurology and related clinical specialties" [68]. NeurologyPocketbook runs on a modern software-as-a-service platform called Notion [69]. The notion is a new web application that aims to make collaborative work easier in the new virtual world. This preserves the Wiki model of collaboration but adds the authenticity of peer review, as shown in Figure 3. This collaborative and peer-reviewed source of medical knowledge is the future wave where knowledge is consumed, created democratically, and updated regularly. Google Meet meetings are held monthly, and a Discord server is set up to enhance communication between authors and faculty. Students do not just learn neurology but advanced digital tools alongside clinical education. The vision of NeurologyPocketbook is beyond medical education and will include health education, where patient education material both in print and video-based format will be collaboratively made [68].

Evolution of MOOCs in Medicine - Mini-Fellowships

MOOCs provided a great opportunity to deliver information-dense lectures to students. Students can replay lectures as many times as needed. Online lectures are much more interactive, e.g., online quizzes, and can help medical students practice their clinical information numerous times and increase their understanding. In 2021 alone, over 40 million people signed up for at least one MOOC, 7% of which were in the field of health and medicine [70]. The data suggests that online medical education is as beneficial, if not more, than offline learning, with the added benefit of mastering one's skills and overcoming weaknesses at one's own pace [71].

One such early example was the Mini-Fellowship in Movement Disorders launched in 2017 by one of the authors (DB) as a six-month-long online fellowship. This is "a program intended to enhance Movement Disorders (MD) training for General Neurologists, Psychiatrists, Physiatrists (PM&R), and Primary Care Providers to improve their skills in Movement Disorders" [72,73]. The program was initiated to fill the vacuum of untrained neurologists in under-developed countries like Pakistan cost-effectively and has since turned into a project of informational exchange between America and the rest of the world. The fellowship uses virtual learning, online modules, and informative feedback through clinical case discussions and has successfully graduated 81 Physicians (mostly neurologists) from 13 different countries.

Limitations and challenges

A review done by Doherty et al. to assess the barriers and solutions to online learning in medical education outlined time constraints, poor technical skills, a lack of infrastructure, poor communication, and deficiency of institutional strategies and support as major limitations to the augmentation of digital neurology [74]. Keeping in mind all these limitations, institutes need to teach technical skills to educators as well as learners. Similarly, policies and approaches to deal with student engagement and fair assessment tests should be taken into consideration.

The transition to Education 4.0 faces two big challenges: 1) Maintenance of authenticity and 2) User Adoption. To maintain an open-access user-generated system that is also authentic will be a huge challenge. This can be overcome with newer AI technologies that automatically flag information that needs to be reviewed. Once users are assured of the safeguards of authenticity and, more importantly, their own ability to flag resources for review, it will bring much-needed confidence in the system and improve adoption.

## Conclusions

As everything is being digitized, we are witnessing the dawn of the "digital clinician." Medical education needs to evolve rapidly to meet the needs of digital native students and providers. A systemic design approach built on the foundation of Industry 4.0 will benefit the current system. This approach has to incorporate our new cyber-physical presence. This is a daunting but necessary task, as the demands of an increasingly digital world have to be met.

This paper demonstrates the efficiency and interconnectedness of the world that is about to be built based on the tenets put forth. It results from the collaboration between authors spanning three different countries and time zones, brought together by digital tools. The paper also almost entirely cites OA literature, without which none of our work would have been possible. The authors highly recommend that institutions and other non-traditional educational startups partner up and collaborate in our new global virtual society. The use of such forces with the aid of technology can advance the study and training of neurology into the future. Healthcare workers can look forward to a new era in medical education that is open access, collaborative, decentralized, authentic, mobile-ready, delivered virtually and in virtual environments, and accessible universally.
